# Analyzing Trends in Suicidal Thoughts Among Patients With Psychosis in India: Exploratory Secondary Analysis of Smartphone Ecological Momentary Assessment Data

**DOI:** 10.2196/67745

**Published:** 2025-05-29

**Authors:** Ameya P Bondre, Aashish Ranjan, Ritu Shrivastava, Deepak Tugnawat, Nirmal Kumar Chaturvedi, Anant Bhan, Snehil Gupta, Abhijit R Rozatkar, Srilakshmi Nagendra, Siddharth Dutt, Soumya Choudhary, Preethi V Reddy, Urvakhsh Meherwan Mehta, John A Naslund, John Torous

**Affiliations:** 1 Sangath, 106, Good Shepherd Colony, Kolar Rd, Bhopal, Madhya Pradesh, 462042, India, 91 8874041444; 2 Department of Psychiatry, All India Institute of Medical Sciences Bhopal, Bhopal, Madhya Pradesh, India; 3 Department of Psychiatry, National Institute of Mental Health and Neurosciences, Bengaluru, Karnataka, India; 4 Department of Global Health and Social Medicine, Harvard Medical School, Boston, MA, United States; 5 Division of Digital Psychiatry, Beth Israel Deaconess Medical Center, Boston, MA, United States

**Keywords:** schizophrenia, suicidality, ecological momentary assessments, India, trends, suicidal, suicidal ideation, suicides, suicidal thoughts, EMA, mobile apps, exploratory study, psychosis, smartphone, tertiary hospitals, outpatients

## Abstract

**Background:**

India has the world’s largest number of suicides, but there is little research on the trends in suicidal thoughts, especially for individuals with psychosis. More research is necessary to develop preventive interventions. Smartphone-based ecological momentary assessments (EMAs) can assess dynamic symptoms, but most EMA studies are conducted in higher income settings and have shorter (≤1 month) follow-up periods.

**Objective:**

This study aimed to examine the duration of onset to offset of suicidal ideation (SI) in tertiary hospital outpatients with psychosis in India.

**Methods:**

This study is an exploratory, secondary analysis of smartphone EMA data nested within the ongoing “Smartphone Health Assessment for Relapse Prevention (SHARP)” project. Tertiary hospital outpatients (n=50) with early course schizophrenia at 2 socioculturally different sites in India were recruited and given the “mindLAMP” app for monitoring mood through daily EMA surveys. The mood survey matched the 9-item Patient Health Questionnaire; the ninth item was used to define an instance of SI (score ≥1). A total of 14 patients with ≥1 SI instances who met the site-specific EMA survey use cutoff were included. We examined the between- and within-person variability in SI and computed the timescale of “episodic” SI (sequences of consecutive daily observations of SI score ≥1). Positive and Negative Syndrome Scale (PANSS) was used to assess changes in psychosis symptoms and its relationship with the temporality of SI.

**Results:**

Over approximately 11 (SD 2.1) months of EMA reporting on average, 3253 mood surveys were filled by the 14 participants (median 213, IQR 147‐256). A total of 521 instances of SI were reported. Monthly SI instances showed substantial within- and between-person variations. Timescale summary statistics revealed episodic SI patterns in 11 patients, with an average of 5.9 episodes (SD 4.4; range:1‐14; n=65) with an episode lasting on average 2.5 days (SD 1.5; range:1‐5.3; n=27). There was an average lag of approximately 59, 66, and 81 days between the time of the first drop in PANSS positive, negative, and general psychopathology scores, respectively, and the last reported SI instance. Results after imputation of missing data showed an average of 12.1 episodes and 228 days (average lag) between the first drop in PANSS scores and last reported SI. This indicated that SI was an enduring vulnerability subsequent to the beginning of clinical improvement in psychosis.

**Conclusions:**

Our study adds to the much-needed evidence base in India to measure the dynamics of suicidal thinking within an individual, for more targeted preventive interventions. Further steps in EMA research are highlighted such as the use of higher frequency “burst” surveys to assess the duration of an SI episode in hours or minutes, and inclusion of both active and passive SI markers to measure the timescale of suicidal thinking.

## Introduction

India has the world’s largest number of suicidal deaths [1], and for every death by suicide in India, there are more than 200 people with suicidal ideations (SIs) [2]. Suicide is a common cause of premature mortality among people living with schizophrenia [3,4] and a recent systematic review has reported a point prevalence of nearly 30% of SIs in people with schizophrenia [5].

Most of the empirical research on the risks of suicidality includes cross-sectional or retrospective studies that distinguish the characteristics between people who experience SIs or suicidal behavior, and those who do not [6-8]. This is true for Indian hospital-based studies on suicide in schizophrenia [9,10]. Longitudinal studies (without ecological momentary assessments [EMAs]) have also mostly examined the prevalence of, and factors associated with suicidal thoughts and behaviors among individuals with early course schizophrenia, as illustrated in a systematic review of 17 studies [11]. There is some evidence of trajectories of SI such as “low-decreasing,” “frequent-stable,” and “frequent-increasing” identified among patients with first-episode psychosis, but these studies do not involve frequent assessments (like in EMAs) and the long-duration prospective study designs involve wider-spaced follow-ups (eg, yearly) [12]. EMA studies involving participants with psychosis also often assess the relationships between SI and other relevant variables such as perceived burdensomeness and thwarted belongingness [13]; or social approach, functioning, or isolation in schizophrenia [14]. With respect to temporality of SI in longitudinal designs, a recent review of 26 studies has demonstrated that intensive time sampling approaches are highly capable in detecting SI at daily and hourly increments [15], but this emerging evidence is not specific to patients of psychosis [15,16]. Specifically, we know little about the temporal dynamics of SI in psychosis, in particular how SI changes or evolves over time (minutes, hours, or days) in an individual with psychosis, or when this individual is at risk [7,17], which is critical to understand how suicidal behavior develops, and design preventive interventions [18]. One key aspect is the duration of onset to offset of an episode of suicidal thinking [18]. Smartphone-based EMAs can assess dynamic symptoms such as SI, and help measure its duration, while enabling a safe disclosure of SI without face-to-face contact with the researcher or clinician [19,20]. However, most EMA studies on suicidality, as also EMA studies on day-to-day functioning of people with schizophrenia have been conducted in higher-income countries [21]. Furthermore, the available EMA studies have sampling periods typically lasting for ≤1 month [20], which is inadequate to inform the estimation of the evolution of SI during longer outpatient care periods in India, reducing the clinical use.

This study aimed to explore the feasibility of using EMA to track SI in tertiary hospital outpatients with psychosis in lower-resource settings in India, duration and temporal trends of SI, particularly the estimation of the duration of onset to offset of SI (primary objective) over a substantially long follow-up period, and association between temporal trends of SI and symptoms of schizophrenia.

## Methods

### Setting

The parent study was conducted at Beth Isreal Deaconess Medical Centre, Boston, United States; Sangath and All India Institute of Medical Sciences (AIIMS) in Bhopal, Madhya Pradesh, India; and National Institute of Mental Health and Neurosciences (NIMHANS) in Bengaluru, Karnataka. This study is a secondary analysis of data from the latter 2 sites in India. Sangath and the AIIMS Bhopal implemented the parent study in Bhopal. Sangath was founded in 1996 and is a leading mental health research nongovernmental organization in India. Since 2011, Sangath has been working closely with the health system in Madhya Pradesh, to advance research efforts aimed at implementing evidence-based mental health services in primary care settings. AIIMS Bhopal is a premier tertiary medical center and a national center of excellence in medical education, biomedical research, and service delivery. In Madhya Pradesh, AIIMS Bhopal represents a leading regional institution for provision of psychiatric services, including the treatment and management of schizophrenia spectrum disorders. NIMHANS is a tertiary care teaching facility that caters to about 400 daily outpatients with an approximately 600-bed psychiatric in-patient facility. Individuals with schizophrenia receiving treatment at NIMHANS comprise a mix of patients having first episode and chronic disease, coming from urban and surrounding rural localities, and those with difficult-to-treat symptoms, referred from across the country.

### Procedures

This study is an exploratory, secondary analysis of smartphone EMA data nested within the ongoing “Smartphone Health Assessment for Relapse Prevention” (SHARP) project or the parent study [22-24], which is guiding the systematic development and adaptation of an open-source smartphone app and digital dashboard—Learn, Assess, Manage and Prevent (LAMP) across diverse cultures and contexts to promote patient-centered care [22-24]. This analysis focuses on the EMA reporting period from September 2021 to April 2023, of outpatients diagnosed with early course schizophrenia at AIIMS in Bhopal in collaboration with Sangath (central India), and NIMHANS in Bengaluru (southern India). The participant sample for the secondary analysis was drawn from the 25 hospital outpatients in each of these 2 parent study sites, recruited during routine outpatient services. Data from the parent study site in Boston, that is, Beth Israel Deaconess Medical Centre was not included for this analysis, as we focused on lower-resource settings in a low- and middle-income country.

Each participant had an initial intake visit and monthly follow-up visits at each site. Participants were recruited from among the clinic outpatients at NIMHANS and AIIMS hospitals. Inclusion criteria of the parent study were the diagnosis of a psychotic spectrum disorder, or a report of symptom onset for psychosis, within the 5 years before the beginning of data collection in 2021, age 45 years or younger, residence in India, and sufficient proficiency in English or Kannada (in Bengaluru) or English or Hindi (in Bhopal). Further details of the parent study procedures have been published in previous studies [22-24].

During the intake visit in the parent study, the participants were provided a detailed study description followed by written informed consent, and then guided to download the smartphone app, mindLAMP 2, from the App store (iOS) or Play store (Android), and provided a walkthrough of the app and the necessary settings [22-24]. The study team provided a smartphone for patients who did not have it. After the app setup, clinical symptoms for psychosis were assessed using the Positive and Negative Syndrome Scale (PANSS; refer to Measures section), in addition to other parent study baseline assessments [22]. PANSS was readministered at each monthly clinical follow-up, in person or remotely, depending on the latest COVID-19 safety guidelines implemented at the sites. Over the postrecruitment period, participants received daily EMA notifications on their mindLAMP app, including EMA surveys, cognitive games, psychoeducation resources, and relaxation exercises. Daily scheduled activities appeared on the app’s feed page, and participants were encouraged to complete the activities daily. In the parent study, mood, anxiety, psychosis, sleep, social functioning, and medication adherence (EMA) surveys were randomized such that 2 of the 6 surveys were sent to participants twice per day, to be completed at least once. Participants reported their mood at the time of the survey prompt.

EMA mood surveys (Patient Health Questionnaire-9 [PHQ-9]), the focus of the exploratory analysis, were sent as part of a “group,” with other EMA surveys, during the morning and evening; they remained “open” for the 12-hour duration or till the next “group” of surveys was assigned. These surveys were used to reduce recall bias and increase the ecological validity of the findings [25], while complementing the monthly psychiatric follow-up assessments, for which the patient attended the hospital in person. Importantly, participants could also access the mood survey and submit the data at any time by directly filling in the survey in the mindLAMP’s “ASSESS” section; especially, if they missed the survey prompt or could not fill the survey via the notification. We expected the participants to fill a minimum of 1 survey daily, to assess the survey “usage” (we have defined “usage” as discussed subsequently). The mood survey matched the PHQ-9 [26] used for screening depression.

### Measures

An instance of SI was measured using the PHQ-9, ninth item score at the EMA prompt (“thoughts that you would be better off dead, or of hurting yourself,” cutoff≥1, range: 0-3) [26]. To assess the relationship between the temporality of SI instances and changes in symptoms of psychosis, we used the PANSS positive, negative, and general psychopathology scales, each rated from 1=absent to 7 points (extreme symptoms). The score range is 7‐49 for the positive and negative scales, and 16‐112 for the general psychopathology scale [27]. PANSS was administered monthly (in person in Bhopal and in person or remotely in Bengaluru) by the site teams.

Erbe D et al [28] compared the interformat reliability of the paper-and-pen version with a computerized version of the PHQ-9 in a clinical sample and obtained comparable internal consistency between the computer (*α*=.88) and paper versions (*α*=.89), and highly significant correlations between the formats (*r*=0.92). The PANSS is a widely used and valid instrument for the assessment of symptom severity in schizophrenia; test-retest reliability for the total score and subscales is reported as 0.77‐0.89 [27,27].

We want to clarify that mood, anxiety, psychosis, sleep, social functioning, and medication adherence (EMA) surveys were randomized such that 2 of the 6 surveys were sent to users twice per day (or total 2 EMA survey prompts), to be completed once (in the parent study). Therefore, the mood survey (PHQ-9) was not sent daily due to random selection. However, participants could access the mood survey from the “ASSESS” section of the mindLAMP app at any time. Therefore, we have considered survey “usage” as the measure of feasibility, instead of “compliance” to survey notifications. By defining a day’s usage as a “minimum of 1 mood survey filled daily, and counting the first survey of the day (in case of multiple surveys filled),” we have accounted for the option that the participants had, that is, to directly access the mood survey in the app. This is consistent with the fact that while the participants may have missed survey notifications, the research team members tracked their app usage on the dashboard and made touch-base calls with participants to encourage them to use the app and fill the surveys (calls made every 2‐3 days as a practice, or depending on observed usage on the dashboard).

### Sampling and Participants

Out of the 50 outpatients (25 at each site) originally sampled for the parent study (convenience sampling), 10 patients in Bhopal and 12 in Bengaluru had more than 1 instance of SI, essential for conducting the intended analysis, and out of these, 6 patients in Bhopal showed a 40% or more daily usage rate, and 8 patients in Bengaluru showed 20% or more daily usage rate on EMA surveys (usage rate: percentage of mood surveys completed of the total surveys expected to be completed daily ie, minimum 1 survey per day over the reporting period). We defined different usage rate cut offs for the 2 sites after considering the average usage rates (56.3%, IQR 46.7%-64%, SD 13.1% for Bhopal and 34%, IQR 24.2%-43.3%, SD 11.8% for Bengaluru), and the substantial variance between the sites in the distribution of survey usage across participants. Missing data in the sampling process included the number of days when the survey was not filled for which we used an imputation model (discussed subsequently). Based on recent compliance rates of 44% for typical EMA surveys and 35% for high-frequency “burst” surveys over a 42-day monitoring period [18], we defined conservative EMA usage cutoffs, given our much longer data reporting period.

### Data Analysis

**Figure 1.**
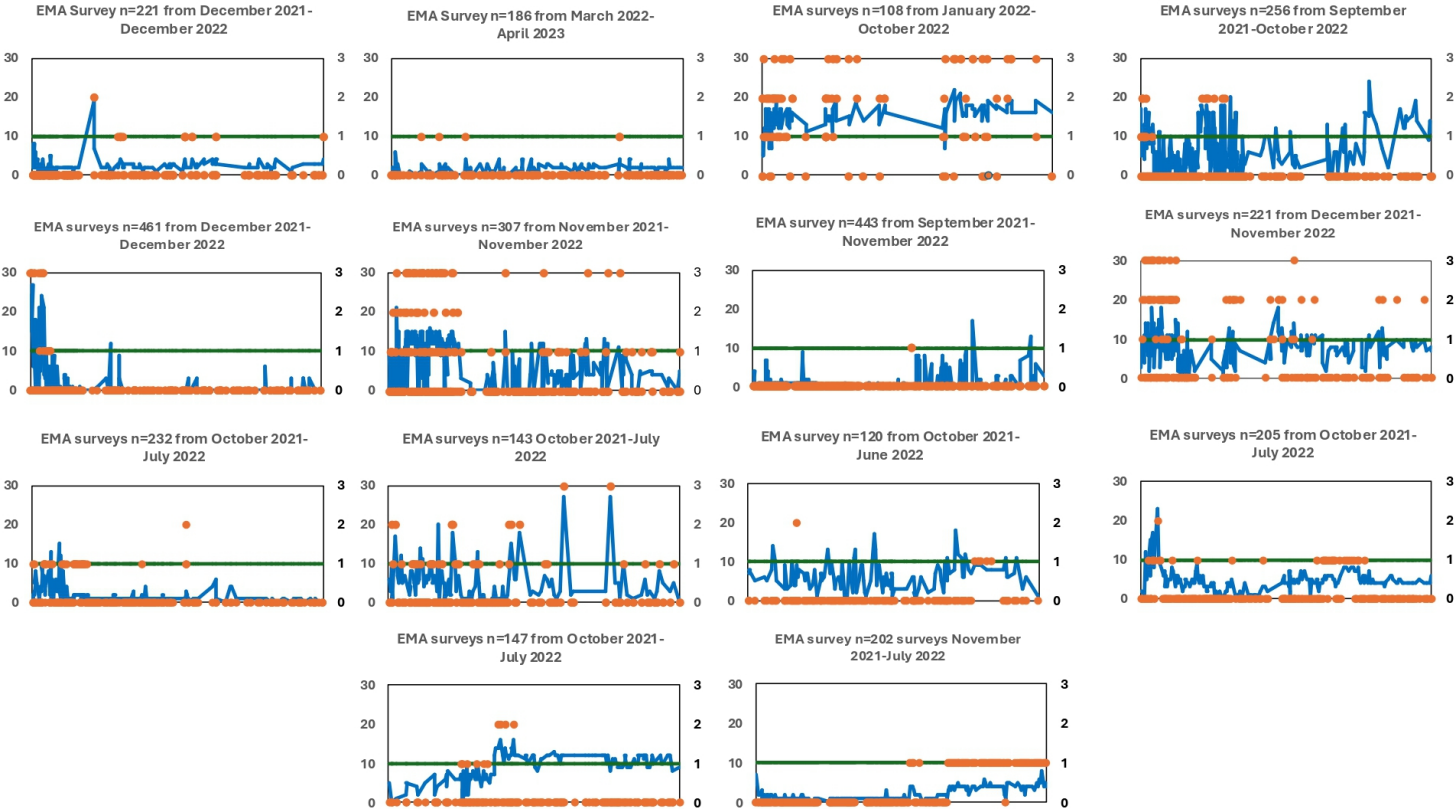
Time series plots of instances of suicidal ideations (PHQ-9 ninth item scores) of individual participants. This chart shows the time-series plots of the 14 total participants across the 2 sites. The blue line represents the total PHQ-9 mood scores (range: 0‐27) plotted on the left-hand axis and the orange dots represent scores of suicidal ideation represented by the PHQ-9 ninth item score ranging from 0 (absent, or “resting”) to 3, graded on the right-hand axis. The gray threshold line represents the PHQ-9 total score cutoff of 10 for moderate depression. The total surveys filled by each participant with the corresponding reporting period are mentioned at the top of the time-series plot. EMA: ecological momentary assessment; PHQ-9: Patient Health Questionnaire-9.

We referred to analytical plans of similar studies that used EMA [18,22] and higher frequency “burst” surveys [18] to map the duration or timescale of SI. All analyses were conducted in Stata (version 14; StataCorp) [29] and Microsoft Excel 2010. First, we examined the mean EMA reporting period and survey usage rates (mean, SD, and IQR) across the 14 patients. Second, we examined the descriptive properties such as mean number of instances of SI, duration (days) between first and last reported SI, percentages of modal responses ie, score of 0 on the ninth item of PHQ-9 or “resting state,” average duration between 2 successive SI instances, and “no reported SI” period that is, days between last SI and last EMA date. Third, we used timescale summary statistics to quantify the rate at which self-reported SI changed over time within individuals. Through visual inspection of each individual time-series (Figure 1), we identified that most patients showed an “episodic” pattern of SI reporting. “Episodic” time-series include distinct periods of elevated (nonzero) responses interspersed by sequences of zeros, which we can interpret as episodes of heightened suicidal thinking. We quantified the rate of change in the time-series by calculating the frequency and duration of episodes of elevated suicidal thinking. We categorized sequences of consecutive daily observations that show SI score ≥1 as episodes; we calculated the duration of an episode by observing how much time elapses before the next occasion (day) when SI is back to its resting state (score=0). The estimated duration was, therefore, an upper bound of the true episode length, as participants may have returned to “normal” EMA response, before the next instance of SI. In addition, we assessed the duration between the date of the first “drop” in PANSS score (marker of the beginning of clinical improvement) and last reported SI instance, to check for a relationship between clinical changes in symptoms of psychosis and temporality of SI.

We acknowledge the challenge of missing data in EMA studies, where engaging with the app and providing symptom data through surveys can have substantial missing values, particularly in real-world settings such as clinic outpatients (as in this study), over a long follow-up period. Therefore, we have used the “Multiple Imputation for Categorical Time Series (MICT) algorithm,” introduced by Halpin (2016), which handles missing data gaps, for the typical form of missing data in longitudinal datasets, by imputing them recursively from their edges [30,31]. MICT model for imputation was originally developed using a multinomial model, and to impute a gap of missing values, the algorithm includes past or future time points. Due to our small participant sample, we could not include covariates (eg, illness duration or gender) in the model, however, we have computed the average frequency of episodes of SI and the average episode duration after imputation, which is the primary aim of the study, and compared these metrics with the timescale summary statistics, which are unadjusted for missing values. Table S1 in Multimedia Appendix 1 includes the detailed data on frequency and duration of SI episodes after imputation for each model, per participant.

### Ethical Considerations

Ethical approval for both study sites was granted by their respective institutional review boards (IRBs): Sangath IRB and AIIMS Bhopal Institutional Human Ethics Committee, and NIMHANS IRB, in accordance with relevant ethical guidelines for human research. All participants in the parent study provided written informed consent before participation, which allowed the use of deidentified data in secondary analyses without additional consent. To protect participants’ privacy and confidentiality, all data were deidentified (a study ID was assigned to each participant), no personally identifiable information was included in the final dataset, and all published data were presented in aggregate form. Participants’ data (eg, case record forms, information sheets, and consent forms) were stored in a locked cabinet in the respective site’s research office. During the study, only deidentified data were used, and the data were only accessible to the research team. There is no identification of individual participants or users in any figures of this paper or its supplementary material. Participants received compensation of INR ₹500 (approximately US $7) for each assessment session in which they participated.

## Results

### Participant Overview

**Figure 2.**
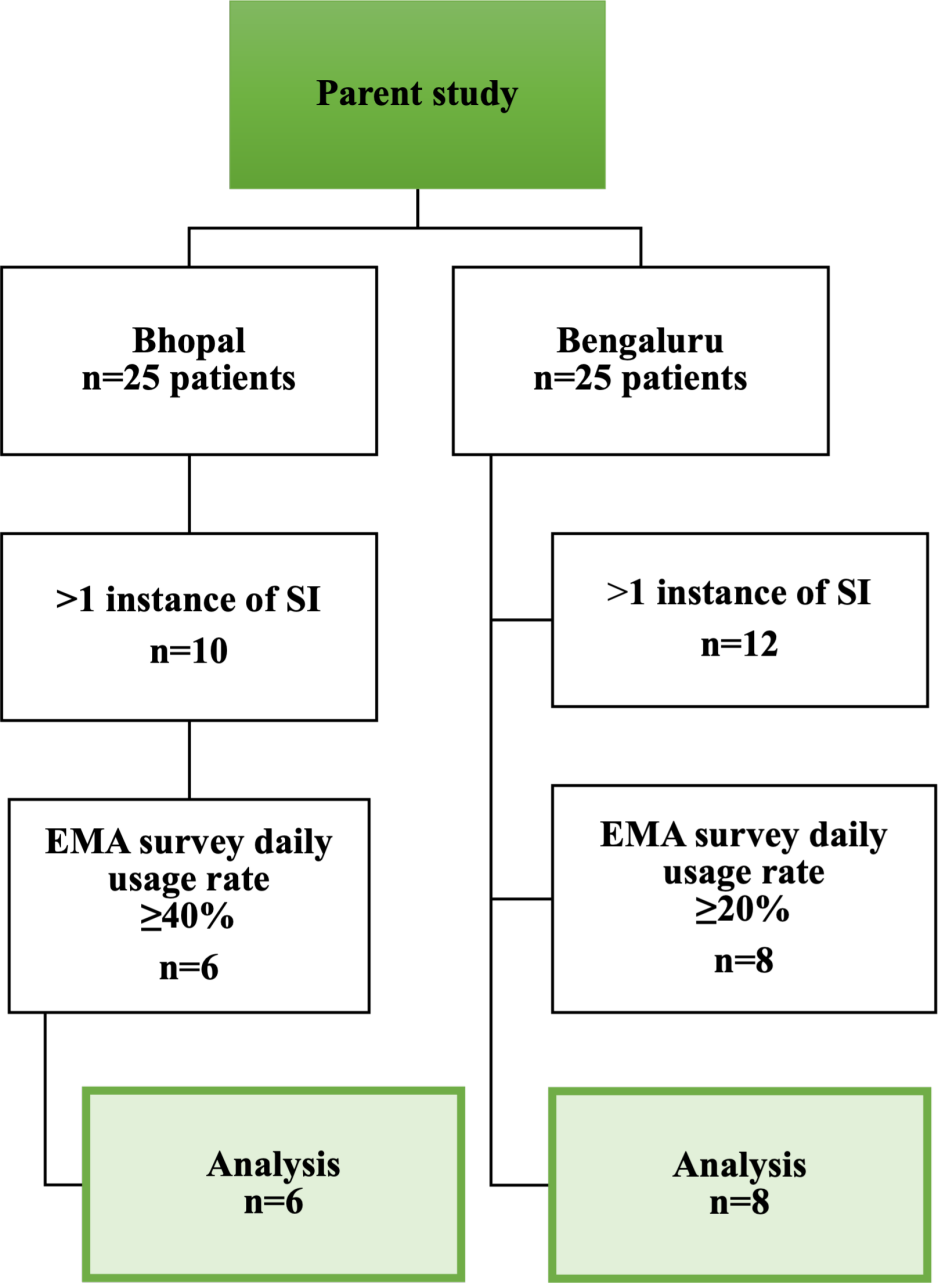
Sample selection for the secondary analysis. The parent study included a cross-sectional sample of 50 patients from Bhopal and Bengaluru sites. A total of 22 patients reported more than 1 instance of suicidal ideation (SI), which was necessary for their inclusion in the secondary analysis, and furthermore, 6 patients from Bhopal and 8 patients from Bengaluru had daily mood survey usage rates, that is, ≥40% and ≥20%, respectively. These 14 patients were included for the final analysis. We defined different usage rate cutoffs for the 2 sites after considering the average usage rates (56.3%, IQR 46.7%-64% for Bhopal and 34%, IQR 24.2%-43.3% for Bengaluru), and the substantial variance between the sites in the distribution of survey usage across participants. EMA: ecological momentary assessment.

Following is the summary of demographic characteristics of the subgroups of patients included in the analysis (n=14 as shown in Figure 2): Bhopal (n=6, average age: 30.8, SD 6.6, range: 23‐42 years; n=3, 50% female; mean 11.8, SD 1.8 years of education; n=5, 83.3% were single); Bengaluru (n=8, average age: 33.9, SD 3.8, range: 31‐42 years; n=3, 37.5% female; mean 13.6, SD 2.8, years of education; n=7, 87.5% were single). We also compared the demographic characteristics between the full (n=22) and the analytic sample (n=14) but found marginal differences (Tables S2 and S3 in ).

### Timescale of Suicidal Ideation

**Figure 3.**
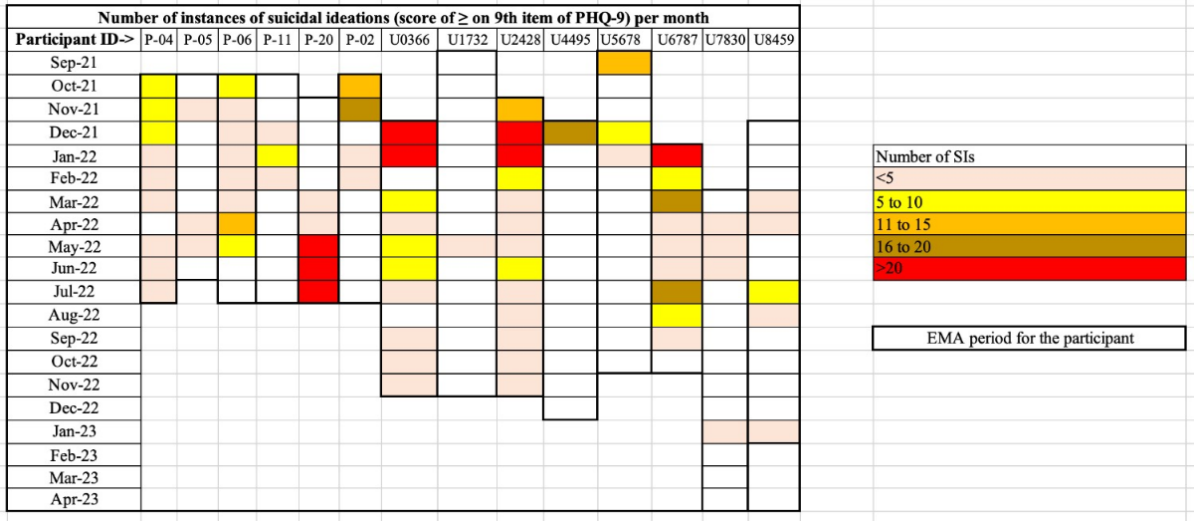
Within and between-person variations in number of SI instances in each month of the reporting period. The grid below represents the participant-wise and month-wise instances of SI (score of 1 or more). The frequency of SI instances has been graded in the legend. The thicker black borders of the individual participant columns indicate the boundaries of the reporting period during which EMA data were collected. April 2023 was the last month of data collection; maximum reporting period was 15 months. EMA: ecological momentary assessment; PHQ-9: Patient Health Questionnaire-9; SI: suicidal ideation.

For the 14 patients included in the secondary analysis, the average days of EMA were 326 or ~11 (IQR 9-13) months, with an average of 56.3% (IQR 46.7%-64%) of EMA survey usage rate for Bhopal and 34% (IQR 24.2%-43.3%) for Bengaluru. In total, 3253 mood surveys were filled by the 14 participants (average: 232 surveys , IQR 147-256) with a total 521 instances of SI, and 76 missing values on PHQ-9 ninth item across the filled surveys (2.3%). The number of SI instances in each month over the reporting period showed substantial within and between-person variations as shown in the grid in Figure 3.

The descriptive analysis (Table 1) of raw data (n=14 patients), disregarding the episodic patterns of SI, revealed high SDs for the average number of SI instances (mean 37.2, SD 34.2), average days between first and last reported SI (mean 188, SD 118.3), percentage of modal (“resting,” score=0) response (mean 78.9%, SD 23.4%), average days of “no reported SI“ period after last reported SI (mean 95.5, SD 114.6) and average days between 2 successive SI instances (mean 12, SD 17.8). Table 2 presents these statistics after imputation of missing data.

**Table 1.** Descriptive statistics of instances of suicidal ideation (SI) reported during ecological momentary assessment (disregarding the episodic pattern of SI and before imputation). Instance of SI is defined as a score of 1 or higher on the ninth item of Patient Health Questionnaire-9; a score of 0 is the “modal” (resting) response. The results in this table do not account for missing values or days with no survey forms received or no ninth item filled in the mood survey.

Characteristics^a^	Statistical values		
	Mean (SD)	Median (IQR)	Range
Number of SI instances	37.2 (34.2)	24.5 (10-70)	2‐106
Days between first and last reported SI	188 (118.3)	192.5 (112-284)	0‐365
Percentage of modal (“resting,” score=0) response	78.9 (23.4)	88.8 (65-95)	15‐99.3
Duration (days) of “no reported SI” period, after last reported SI	95.5 (114.6)	53 (6-157)	0‐359
Duration (days) between successive SI instances^b^^,c^	12 (17.8)	5.2 (2.8-7.9)	0‐65

a Spearman correlation between Patient Health Questionnaire-9 total and SI score at ecological momentary assessment prompts was 0.47 (n=3176, *P*<.001); correlation between Patient Health Questionnaire-9 score>=10 and suicidal ideation score ≥1 was 0.32 (n=245, *P*<.001).

b Disregarding the episodic pattern.

c To understand the difference between “days between successive SI instances” and “episode duration” (refer to the episode data in Table 3), let’s take an example of a patient who has 2 suicidal ideation over 2 consecutive days; the average duration between the successive suicidal ideations is 1 day and the episode duration is also 1 day. However, if there are 4 suicidal ideations over 4 consecutive days, the episode duration is 4 days but the average duration between suicidal ideations is 1 day.

**Table 2.** Descriptive statistics of instances of SI^a^ reported during ecological momentary assessment (disregarding the episodic pattern of SI) after imputation. The results in this table have been calculated after imputation, and account for missing values, which explains the differences in the means, compared with Table 1.

Statistics	Mean (SD)	Median (IQR)	Range
Number of SI instances (n)	70.8 (67.4)	43.7 (23.1-87.5)	10.8‐224.7
Days between first and last reported SI (days)	296.6 (54.8)	287.3 (266.85‐344.45)	212.2‐367.9
Percentage of modal (“resting,” score=0) response (%)	76.8 (23.2)	84.5 (65‐91.9)	17.1‐97.2
Duration of “no reported SI” period, after last reported SI (days)	10.7 (11.1)	9.7 (1.2‐19.4)	0‐34.2
Duration between successive SI instances (days)	11.3 (13.5)	6.4 (2.9‐12.2)	1.2‐51.9

a SI: suicidal ideation.

As the SI patterns across patients were largely episodic (except for 3 patients, which showed highly variable distribution of SI), the mean duration quantities showed high SDs in the raw data (Tables1  2). Therefore, conducting the descriptive analysis at the episode-level was important, which revealed less noise even with a smaller sample (n=11, refer to Tables3  4). Timescale summary statistics revealed an average of 5.9 episodes (range: 1‐14, SD 4.4, 65 total episodes) with an episode of heightened suicidal thinking lasting for 2.5 days on average (range: 1‐5.3, SD 1.5, 27 total days). Please refer to Tables3  4 for these results.

**Table 3.** Timescale summary statistics to calculate the frequency and duration of episodes of SI^a^ (before imputation). Episodes are categorized sequences of consecutive daily observations that show SI score ≥1. We calculated the duration of an episode by observing how much time elapses before the next occasion (day) when SI is back to its resting state (score=0). The estimated duration was, therefore, an upper bound of the true episode length, as participants may have returned to “normal” ecological momentary assessment response, before the next instance of SI.

Statistics	Mean (SD)	Range	Sum	IQR
Frequency of SI episodes (episodes)	5.9 (4.4)	1‐14	65	3‐11
Average SI episode duration (days)	2.5 (1.5)	1‐5.3	27	1.2‐3.2

a SI: suicidal ideation.

**Table 4.** Timescale summary statistics to calculate the frequency and duration of episodes of suicidal ideation after imputation. We conducted the imputation modelling with 13 out of the 14 participants. We excluded one participant who had 2 suicidal ideation instances reported on the same day and no other instance reported throughout their participation, which precluded an estimation of episodes.

Statistics	Mean (SD)		Range	Sum	IQR
Frequency of SI^a^ episodes (episodes)	12.1 (13.2)		1.1‐41.5	157	2.3‐16
Average SI episode duration (days)	2.1 (1.8)		1‐6.3	28	1‐2.1

a SI: suicidal ideation.

In addition, we have also analyzed the survey reporting rate and SI instance reporting rate of participants, site-wise, for a subsample of participants, for 4 different 6-hour time zones. For Bhopal participants, the maximum or 57.4% of the surveys (343 surveys filled) were filled during the 6 AM to <12 PM slot; and SI reporting rate was highest during this slot, ie, 66.7% (104 SI instances). For Bengaluru participants, 53.9% of the surveys (1147 surveys filled) were filled during the 12 PM to <6 PM slot; and SI reporting rate during this slot was 42.6% (141 SI instances).

### Relationship Between Suicidal Ideation and Changes in Symptoms of Psychosis

**Figure 4.**
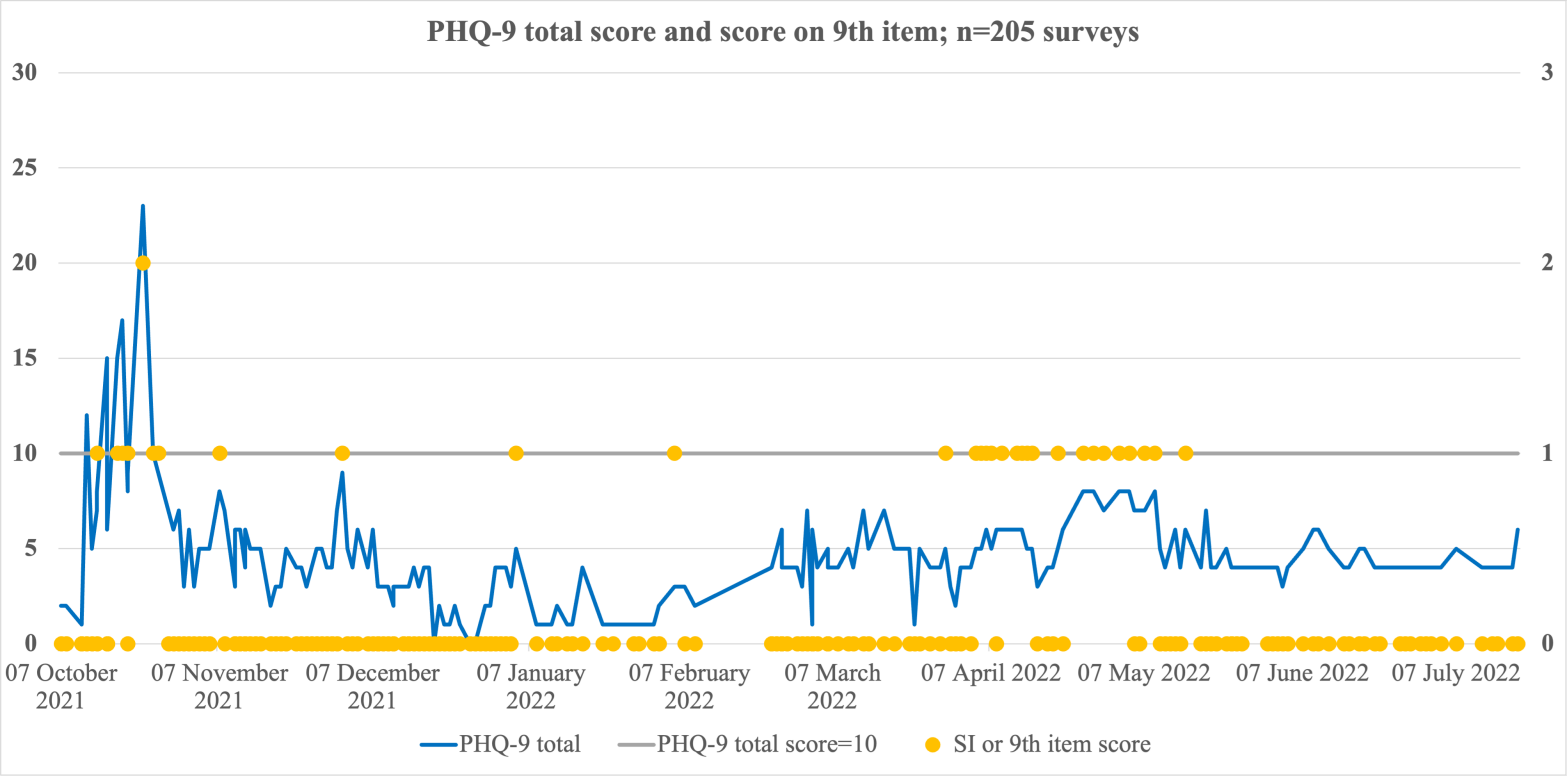
SI time series (ecological momentary assessment mood survey data) of a participant. Figures 4 and 5 aim to respectively show the temporal patterns in SI, and the changes in monthly Positive and Negative Syndrome Scale (clinical in person assessment) scores for the same (illustrated) participant, with available ecological momentary assessment and Positive and Negative Syndrome Scale (in person) assessment data. This figure shows an SI item-level response of 0 (no SI, or modal response), 1 and 2 (scaled on the right-hand axis); the horizontal line passing through 10 marks the SI cut-off of 1 used in this study. Each dot represents the score of SI instance graded from 0 to 3. The graph line indicates the total PHQ-9 score for the same timepoints, scaled on the left-hand axis. An episodic SI pattern is visible with clusters of SI instances (dots) separated by periods of modal response (before imputation). PHQ-9: Patient Health Questionnaire-9; SI: suicidal ideation.

We also conducted an analysis of the relationship between changes in symptoms of psychosis and the temporality of patterns of SI (Figures4  5 refer to the same participant).

**Figure 5.**
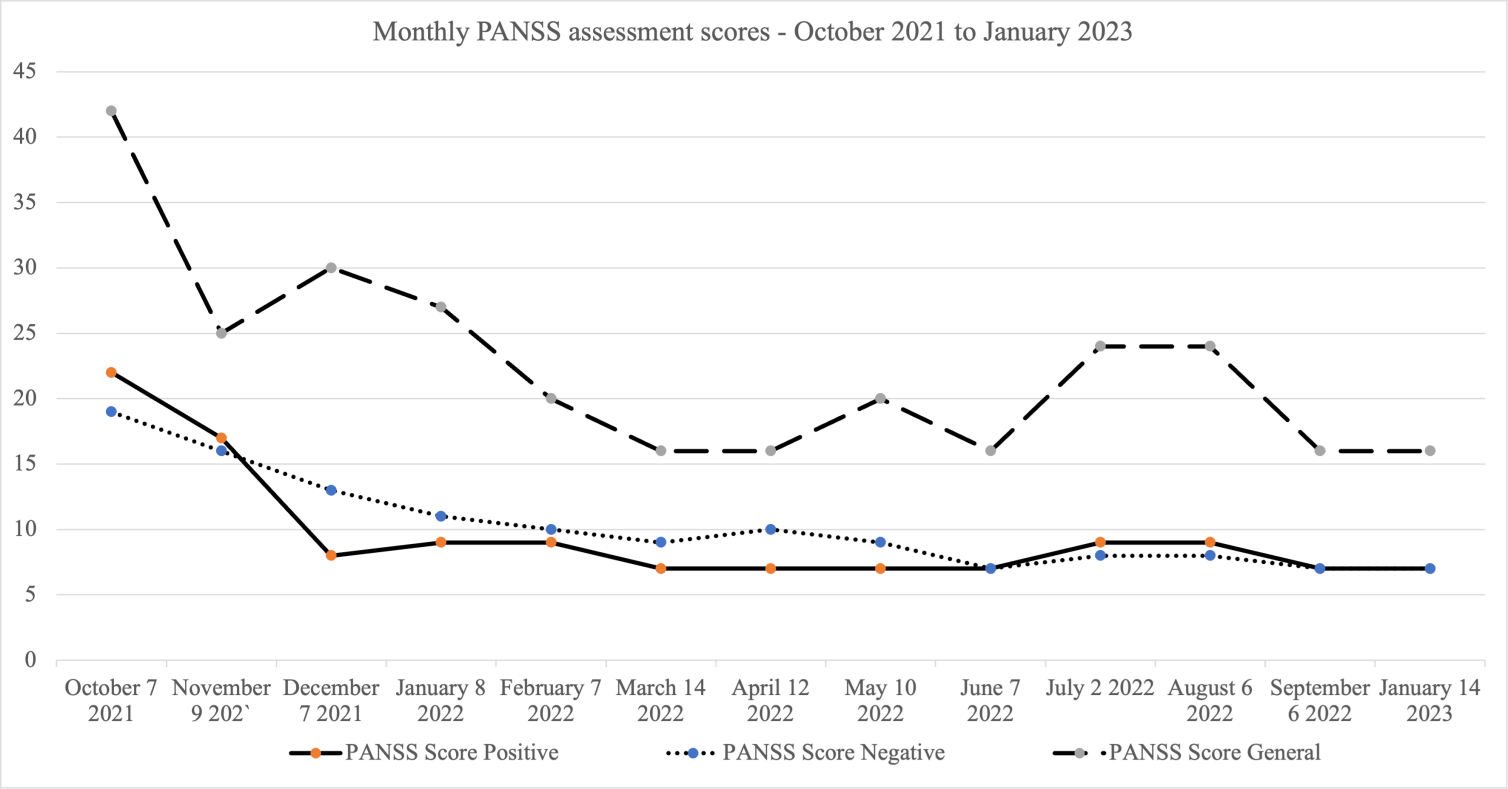
Monthly PANSS scores. This figure shows the monthly changes in positive, negative, and general psychopathology scores for the reporting period, in case of the same participant shown in Figure 4. First drop in PANSS scores was observed in November 2021, but enduring suicidal ideation vulnerability was observed till as late as May 2022 (Figure 4). PANSS: Positive and Negative Syndrome Scale.

Table 5 shows the distribution of PANSS assessments from September 2021 (when EMA surveys also started) to April 2023 (n=14 patients, 154 total assessments, 11 assessments per patient on average [SD 2.1, range 6‐13]).

**Table 5.** Distribution of Positive and Negative Syndrome Scale assessments (monthly), showing the number of assessments conducted, cumulative, and average per participant.

	Number of assessments
Mean (SD)	11 (2.1)
Range	6-13
Sum	154

We examined the number of days that passed between the first drop in PANSS score and the last reported SI, within the PANSS reporting period. As shown in Table 6, there was an average lag of ~59 days, 66 days and 81 days between the first drop in PANSS positive, negative, and general psychopathology score respectively, as a marker of the beginning of clinical improvement, and the last reported SI. Negative values for “days” implied that the drop in PANSS score occurred after the instances of SI, which partly inflated the SDs (n=3 patients), however, in most patients (n=11), the lag was positive indicating that the last reported SI was subsequent to the first signs of clinical improvement on PANSS. Please refer to Table 6 for these results. Table 7 shows these results after imputation of missing data.

**Table 6.** Duration (in days) between the timing of the first drop in Positive and Negative Syndrome Scale (PANSS) score and last reported suicidal ideation (SI), within the PANSS reporting period (before imputation). The results summarize the relationship between first signs of clinical improvement on PANSS and persistence of SI. These results are unadjusted for missing values.

Conditions	Duration (days)		
	Mean SD	Median (IQR)^a^	Range^a^
Between first drop in PANSS positive score and last SI	58.6 (66.7)	85 (–6.5 to 114)	–50 to 132
Between first drop in PANSS negative score and last SI	66 (82.9)	65 (9 to 107)	–57 to 207
Between first drop in PANSS general score and last SI	81 (74.4)	79 (28 to 121)	–40 to 207

a The negative value represents the suicidal ideation incidence before the first drop in PANSS score (for n=3 patients).

**Table 7.** Duration between the timing of the first drop in Positive and Negative Syndrome Scale (PANS) score (in days) and last reported suicidal ideation (SI), within the PANSS reporting period, after imputation. These results are adjusted for missing values, or after imputation.

Conditions	Duration (days)			
	Mean (SD)	Median (IQR)	Range^a^	Frequency, n
First drop in PANSS positive score and last SI	248.2 (47.7)	244.6 (210.4-285.5)	176.6 to 327.4	12
First drop in PANSS negative score and last SI	232.7 (89.4)	242.4 (197.5-293)	–14.55 to 327.4	13
First drop in PANSS general score and last SI	204.8 (112.5)	225.5 (197.5-254.3)	–37 to 337.9	13

a The negative value represents the suicidal ideation incidence before the first drop in PANSS score (for n=3 patients).

## Discussion

### Principal Findings

First, the findings of this study show that it is feasible to collect smartphone EMA data on SI from outpatients having schizophrenia in relatively lower-resource hospital settings in India. This builds on the feasibility and acceptability of EMAs as one of the digital phenotyping methods [16,32] to generate novel forms of data on suicide risk. The demonstrated user engagement in clinical settings in this study is notable (3253 mood surveys filled by 14 patients with schizophrenia over 15 months, with a low proportion [2%] of missing value on the ninth item of PHQ-9), especially as low engagement of health apps as a limiting factor in their regular clinical implementation has been cited in literature [33]. The research team had involved the patients, caregivers, and their attending clinicians in the development and refinements of mindLAMP through earlier extensive focus group discussions in the study sites [34,35], which could have contributed to the greater engagement toward this app to generate clinical data on SI, which was otherwise difficult to obtain in face-to-face models of care where patients usually meet with their attending clinician monthly or every 2 months. It is also important to note that the study could analyze SI data over more than a year of follow-up outpatient visits, representing the typical periods over which patients with psychosis usually follow-up at government hospitals in India, and potentially other similar contexts of the Global South, unlike the much shorter (≤1 month) EMA monitoring periods observed in studies [19]. Related to feasibility, we also found (through reminder or touch-base phone calls to encourage the participants to use the app) that factors affecting EMA usage included low battery-life of the phone due to the passive data collection (eg, GPS data) on the mindLAMP app (beyond the scope of this analysis) and variations in clinical symptoms of schizophrenia potentially affecting app usage. For the latter, we computed Pearson correlations between mood survey usage rate, as defined by a minimum of 1 daily mood survey, and the PANSS score of the previous and the succeeding months. Overall, weak correlation coefficients were observed between survey usage and either the previous or succeeding month’s PANSS score, with somewhat higher and positive correlations noted for the relationship between previous month’s PANSS score and succeeding month’s survey usage rate (succeeding month’s usage and previous month PANSS positive score: 0.26, *P*=.003, n=127 observations; PANSS negative score: 0.01, *P*=.8, n=127 observations; and PANSS general score: 0.18, *P*=.0437, n=127 observations).

Second, the results show substantial within and between person variation in SI, and the attempt to map the timescale of SI among hospital outpatients with schizophrenia is an early effort in the Indian context, to the knowledge of the authors. The initial noise observed in the duration between first and last reported SI and between successive SI instances due to small sample size and episodic SI pattern was controlled by using timescale statistics to help assess the SI evolution better, which revealed that on average, there were 6 SI episodes per patient, each 2.5 days long. Furthermore, with imputation of missing values, 12 SI episodes per patient were reported on average, each 2.1 days long. Therefore, the timescale statistics provide a sense of the intensity of SI as experienced by the patient, and with more rigorous examinations (eg, by high-frequency burst EMA surveys), we can attempt to examine an “episode” in terms of hours.

Third, this secondary analysis and the parent study controlled to the extent possible, the biases that are found in EMA surveys; for example, participants were allowed to postpone an EMA prompt or reject a prompt, which helped reduce practice effects [36], although the statistical significance of the difference in practice effects for specific EMA items was not monitored in the parent study; also, the grid in Figure 3 shows possibly low “reactive” effects because clustering of SI counts of different degrees (color coded in the grid) was seen throughout the EMA period for most patients, and not necessarily in the beginning of the study [37].

Finally, in relation to first signs of clinical improvement in PANSS+, PANSS−, and PANSS general scores, SI instances were observed after approximately 60, 66, and 81 days (approximately 2‐3 months) respectively (within the PANSS reporting period), indicating that SI was an enduring vulnerability even after the beginning of early improvement in positive, negative, and general psychopathology symptoms of psychosis, rather than a short-term crisis [38]. After imputation, the persistence of SI after early signs of clinical improvement was more pronounced; for example, after clinical improvement, SI instances were observed after approximately 248, 232, and 204 days, respectively (approximately 7‐8 months) for PANSS+, PANSS–, and PANSS general scores.

### Limitations

The study has several limitations. First, the PHQ-9 measures passive SI, and it has been shown through rigorous EMA and higher-frequency burst survey analyses that active and passive SI can operate on different timescales [18]. Furthermore, previous evidence states that item-9 of the PHQ-9 is not sufficient to assess suicide risk and suicide ideation, with limited use in certain demographic and clinical subgroups, which requires further research [39]. It was not possible in this study design of a secondary analysis nested in a completed primary study, to understand the frequency, intensity and duration of an instance of SI, or the exploration of active SI. Relatedly (aligned with the study aim), to explore the temporality of SI, we selected a lower cutoff of 1 on the PHQ-9 item-9; but this precluded an analysis of SI severity in relation to time. Second, while the EMA monitoring period in this study was long given the length of outpatient care for persons with psychosis, the observed mean mood survey usage rates of 56.3% (Bhopal) and 34% (Bengaluru) were to an extent, comparable with the mean EMA compliance (44%) observed in a recent assessment of the SI timescale [18], which was a reference for this analysis. There is a caveat though that we have little knowledge on the benchmarks or realistic expectations of what compliance or survey usage rates should be with EMA and higher-frequency sampling. Taking the usage rates together with a low sample size of patients in our study, and the fact that quantitative assessments of “fatigue” effects were not within the scope of the parent study (ie, the change in overall EMA survey usage over time spent in the study), it is challenging to make broad claims around the episodic nature of SI (frequency and duration of episodes) and the relationship of SI with improvement on PANSS. Third, the parent study could not use higher frequency burst surveys due to potential respondent burdens on hospital outpatients, which limited the scope of potentially identifying 19% more patients with SI [18], and rendered the durations of SI episodes in days, and not in hours. This also reduced the ecological validity, and data patterns resembled more of a daily dairy than conventional EMA; though it needs to be recognized that this is a real-world lower-resourced outpatient clinic sample in a long follow-up period. Higher-frequency (hours or minutes apart) sampling is necessary to accurately characterize within-person dynamics of suicidal thinking, although with the costs of respondent burden and potential reductions in overall survey usage. Fourth, “anonymous sampling,” which increases the overall identification of endorsement of rates of SI [40,41] was not done in the parent study , that is , participants had to provide their name, address, or phone number in the process of recruitment in the outpatient clinic. Fifth, the type of population and setting for the above analysis to estimate the timescale of SI limits the generalizability of the findings in multiple ways, for example, (a) the findings may not be generalizable to people living with schizophrenia who are not engaged in outpatient care, or who may be unable or hesitant to seek formal psychiatric outpatient care; (b) the findings may not apply to older people living with the illness given our study age group, as also to younger people in the community (nonhospital) settings, and adults with schizophrenia who are hospitalized; and (c) the data were collected during COVID-19, which may point to unique stressors and the findings therefore, may not generalize to people outside this period of time. Sixth, we want to highlight the small sample size for analysis, that is, 14 of 22 participants with SI had EMA data of sufficient quality that could be analyzed, which limits the feasibility of our approach. Finally, SI or suicidal thoughts (as captured by PHQ-9) are relatively common, and typically not serious unless the individual has a plan, intent, or makes a suicide attempt. This analysis does not have these details, and without knowing the complete suicidal intention, it is difficult to design or inform prevention efforts.

### Conclusions

Even as identifying “when” a person is at risk of suicide is as important as identifying “who” is at risk, and it is recognized that mapping the evolution of suicidal thinking in high-risk individuals such as outpatients with psychosis is necessary to make significant progress in suicide prevention efforts, the national suicide prevention strategy in India [42] only mentions “digital interventions” as short-term prevention strategies. This is because the research on examining the patterns of suicidal thoughts in real time among mental health clinic outpatients in India is extremely scarce. Our study adds to the much-needed evidence base to further highlight the next steps in EMA research on measuring the changes in suicidal thinking, that is, the use of higher-frequency “burst” surveys to assess the duration of an episode of SI in hours or even minutes for more targeted interventions; inclusion of markers of active and passive SI and understanding the shift from passive to active SI, which presents a therapeutic window of opportunity [43], in addition to collecting data on attempt; addition of passive data (eg, mobility markers) to the analysis; and inclusion of other relevant measures (such as PANSS in this analysis) such as changes in anxiety level and sleep to arrive at more precise estimations of the intensity and duration of individual periods of heightened suicidal thinking experienced by an individual.

## Supplementary material

10.2196/67745 Multimedia Appendix 1 Supplemental tables of imputation models for missing data and additional demographic characteristics of participants.

## Abbreviations

AIIMS: All India Institute of Medical Sciences
EMA: ecological momentary assessment
IRB: institutional review board
LAMP: Learn, Assess, Manage and Prevent
MICT: Multiple Imputation for Categorical Time Series
NIMHANS: National Institute of Mental Health and Neurosciences
PANSS: Positive and Negative Syndrome Scale
PHQ-9: Patient Health Questionnaire-9
SHARP: Smartphone Health Assessment for Relapse Prevention
SI: suicidal ideation

## References

[1] Yadav S, K K A, Cunningham SA (2023). Changing pattern of suicide deaths in India. Lancet Reg Health Southeast Asia.

[2] Amudhan S, Gururaj G, Varghese M (2020). A population-based analysis of suicidality and its correlates: findings from the National Mental Health Survey of India, 2015-16. Lancet Psychiatry.

[3] Piotrowski P, Gondek TM, Królicka-Deręgowska A, Misiak B, Adamowski T, Kiejna A (2017). Causes of mortality in schizophrenia: an updated review of European studies. Psychiatr Danub.

[4] Laursen TM (2019). Causes of premature mortality in schizophrenia: a review of literature published in 2018. Curr Opin Psychiatry.

[5] Bai W, Liu ZH, Jiang YY (2021). Worldwide prevalence of suicidal ideation and suicide plan among people with schizophrenia: a meta-analysis and systematic review of epidemiological surveys. Transl Psychiatry.

[6] Fazel S, Runeson B (2020). Suicide. N Engl J Med.

[7] Millner AJ, Robinaugh DJ, Nock MK (2020). Advancing the understanding of suicide: the need for formal theory and rigorous descriptive research. Trends Cogn Sci.

[8] Turecki G, Brent DA, Gunnell D (2019). Suicide and suicide risk. Nat Rev Dis Primers.

[9] Mamtani H, Pathak H, Sakhardande K (2024). Suicide attempts in schizophrenia – a large retrospective cohort study from a tertiary care psychiatry centre in India. Asian J Psychiatr.

[10] Nath S, Kalita KN, Baruah A, Saraf AS, Mukherjee D, Singh PK (2021). Suicidal ideation in schizophrenia: a cross-sectional study in a tertiary mental hospital in North-East India. Indian J Psychiatry.

[11] Sicotte R, Iyer SN, Kiepura B, Abdel-Baki A (2021). A systematic review of longitudinal studies of suicidal thoughts and behaviors in first-episode psychosis: course and associated factors. Soc Psychiatry Psychiatr Epidemiol.

[12] Madsen T, Karstoft KI, Secher RG, Austin SF, Nordentoft M (2016). Trajectories of suicidal ideation in patients with first-episode psychosis: secondary analysis of data from the OPUS trial. Lancet Psychiatry.

[13] Parrish EM, Chalker SA, Cano M (2021). Ecological momentary assessment of interpersonal theory of suicide constructs in people experiencing psychotic symptoms. J Psychiatr Res.

[14] Parrish EM, Chalker S, Cano M (2024). Ecological momentary assessment of social approach and avoidance motivations in serious mental illness: connections to suicidal ideation and symptoms. Arch Suicide Res.

[15] Ammerman BA, Law KC (2022). Using intensive time sampling methods to capture daily suicidal ideation: a systematic review. J Affect Disord.

[16] Butner JE, Bryan CJ, Tabares JV (2021). Temporal-dimensional examination of the Scale for Suicidal Ideation in a cohort of service members in treatment for PTSD. Psychol Trauma.

[17] Sewall CJR, Wright AGC (2021). Personalizing suicidology. Crisis.

[18] Coppersmith DDL, Ryan O, Fortgang RG, Millner AJ, Kleiman EM, Nock MK (2023). Mapping the timescale of suicidal thinking. Proc Natl Acad Sci U S A.

[19] Kivelä L, van der Does WAJ, Riese H, Antypa N (2022). Don’t miss the moment: a systematic review of ecological momentary assessment in suicide research. Front Digit Health.

[20] Sedano-Capdevila A, Porras-Segovia A, Bello HJ, Baca-García E, Barrigon ML (2021). Use of ecological momentary assessment to study suicidal thoughts and behavior: a systematic review. Curr Psychiatry Rep.

[21] Mote J, Fulford D (2020). Ecological momentary assessment of everyday social experiences of people with schizophrenia: a systematic review. Schizophr Res.

[22] Lakhtakia T, Bondre A, Chand PK (2022). Smartphone digital phenotyping, surveys, and cognitive assessments for global mental health: initial data and clinical correlations from an international first episode psychosis study. Digit Health.

[23] Cohen A, Naslund JA, Chang S (2023). Relapse prediction in schizophrenia with smartphone digital phenotyping during COVID-19: a prospective, three-site, two-country, longitudinal study. Schizophrenia (Heidelb).

[24] Rodriguez-Villa E, Mehta UM, Naslund J (2021). Smartphone Health Assessment for Relapse Prevention (SHARP): a digital solution toward global mental health. BJPsych Open.

[25] Shiffman S, Stone AA, Hufford MR (2008). Ecological momentary assessment. Annu Rev Clin Psychol.

[26] Kroenke K, Spitzer RL, Williams JB (2001). The PHQ-9: validity of a brief depression severity measure. J Gen Intern Med.

[27] Kay SR, Fiszbein A, Opler LA (1987). The Positive and Negative Syndrome Scale (PANSS) for schizophrenia. Schizophr Bull.

[28] Erbe D, Eichert HC, Rietz C, Ebert D (2016). Interformat reliability of the patient health questionnaire: validation of the computerized version of the PHQ-9. Internet Interv.

[29] Stata.

[30] Emery K, Studer M, Berchtold A (2024). Comparison of imputation methods for univariate categorical longitudinal data. Qual Quant.

[31] Halpin B (2016). Multiple imputation for categorical time series. The Stata Journal: Promoting communications on statistics and Stata.

[32] Pizzoli SFM, Monzani D, Conti L, Ferraris G, Grasso R, Pravettoni G (2023). Issues and opportunities of digital phenotyping: ecological momentary assessment and behavioral sensing in protecting the young from suicide. Front Psychol.

[33] Torous J, Nicholas J, Larsen ME, Firth J, Christensen H (2018). Clinical review of user engagement with mental health smartphone apps: evidence, theory and improvements. Evid Based Ment Health.

[34] Bondre AP, Shrivastava R, Raghuram H (2022). A qualitative exploration of perceived needs and barriers of individuals with schizophrenia, caregivers and clinicians in using mental health applications in Madhya Pradesh, India. SSM - Mental Health.

[35] Rodriguez-Villa E, Rozatkar AR, Kumar M (2021). Cross cultural and global uses of a digital mental health app: results of focus groups with clinicians, patients and family members in India and the United States. Glob Ment Health.

[36] Wrzus C, Neubauer AB (2023). Ecological momentary assessment: a meta-analysis on designs, samples, and compliance across research fields. Assessment.

[37] Husky M, Olié E, Guillaume S, Genty C, Swendsen J, Courtet P (2014). Feasibility and validity of ecological momentary assessment in the investigation of suicide risk. Psychiatry Res.

[38] Simon GE, Rutter CM, Peterson D (2013). Does response on the PHQ-9 Depression Questionnaire predict subsequent suicide attempt or suicide death?. Psychiatr Serv.

[39] Na PJ, Yaramala SR, Kim JA (2018). The PHQ-9 Item 9 based screening for suicide risk: a validation study of the Patient Health Questionnaire (PHQ)−9 Item 9 with the Columbia Suicide Severity Rating Scale (C-SSRS). J Affect Disord.

[40] Deming CA, Harris JA, Castro-Ramirez F (2021). Inconsistencies in self-reports of suicidal ideation and attempts across assessment methods. Psychol Assess.

[41] Vannoy SD, Andrews BK, Atkins DC (2017). Under reporting of suicide ideation in US army population screening: an ongoing challenge. Suicide Life Threat Behav.

[42] Vijayakumar L, Chandra PS, Kumar MS (2022). The national suicide prevention strategy in India: context and considerations for urgent action. Lancet Psychiatry.

[43] Nahum-Shani I, Smith SN, Spring BJ (2018). Just-in-time Adaptive Interventions (JITAIs) in mobile health: key components and design principles for ongoing health behavior support. Ann Behav Med.

