# Supplementary material for: Analyzing Trends in Suicidal Thoughts Among Patients With Psychosis in India: Exploratory Secondary Analysis of Smartphone Ecological Momentary Assessment Data
Source: JMIR Form Res. 2025 May 29;9:e67745. doi: 10.2196/67745 (PMC12140503; doi:10.2196/67745)
Supplement: Multimedia Appendix 1 [file formative-v9-e67745-s001.docx]

Table S1 - Imputation models for missing data

Note: For each patient (Patient ID/PID) we have tabulated the results after imputation modelling, including the frequency of suicidal ideation episodes, the total number of days of the episodes (or ‘DAYS’) and the average duration of a single episode in days. These metrics have been summarised for each imputation model run as part of the ‘Multiple Imputation for Categorical Time Series (MICT)’ approach.

| PID -> | U5088299196 | | | U1447216773 | | | U3608245527 | | | U4200647802 | | | U5241094593 | | | U1288641052 | | |
| --- | --- | --- | --- | --- | --- | --- | --- | --- | --- | --- | --- | --- | --- | --- | --- | --- | --- | --- |
|  |  | | |  | | |  | | |  | | |  | | |  | | |
| Model # | Frequency of episodes | DAYS | average duration of episode in days | Frequency of episodes | DAYS | average duration of episode in days | Frequency of episodes | DAYS | average duration of episode in days | Frequency of episodes | DAYS | average duration of episode in days | Frequency of episodes | DAYS | average duration of episode in days | Frequency of episodes | DAYS | average duration of episode in days |
| M0 | 6 | 14 | 2.3 | 5 | 8 | 1.6 | 0 | 0 |  | 4 | 8 | 2.0 | 1 | 1 | 1 | 11 | 53 | 4.8 |
| M1 | 11 | 20 | 1.8 | 18 | 29 | 1.6 | 2 | 3 | 1.5 | 12 | 17 | 1.4 | 3 | 3 | 1 | 13 | 68 | 5.2 |
| M2 | 9 | 17 | 1.9 | 15 | 21 | 1.4 | 0 | 0 |  | 5 | 9 | 1.8 | 3 | 3 | 1 | 12 | 67 | 5.6 |
| M3 | 7 | 15 | 2.1 | 14 | 22 | 1.6 | 1 | 1 | 1 | 7 | 15 | 2.1 | 1 | 1 | 1 | 11 | 65 | 5.9 |
| M4 | 8 | 20 | 2.5 | 21 | 39 | 1.9 | 0 | 0 |  | 6 | 11 | 1.8 | 2 | 2 | 1 | 12 | 58 | 4.8 |
| M5 | 9 | 19 | 2.1 | 20 | 27 | 1.4 | 1 | 1 | 1 | 9 | 14 | 1.6 | 1 | 1 | 1 | 12 | 71 | 5.9 |
| M6 | 10 | 19 | 1.9 | 17 | 24 | 1.4 | 1 | 1 | 1 | 8 | 16 | 2.0 | 2 | 2 | 1 | 12 | 66 | 5.5 |
| M7 | 7 | 15 | 2.1 | 18 | 25 | 1.4 | 0 | 0 |  | 7 | 14 | 2.0 | 2 | 2 | 1 | 13 | 66 | 5.1 |
| M8 | 12 | 23 | 1.9 | 18 | 25 | 1.4 | 1 | 1 | 1 | 6 | 10 | 1.7 | 1 | 1 | 1 | 11 | 56 | 5.1 |
| M9 | 7 | 16 | 2.3 | 13 | 21 | 1.6 | 1 | 1 | 1 | 10 | 17 | 1.7 | 3 | 3 | 1 | 13 | 60 | 4.6 |
| M10 | 7 | 16 | 2.3 | 12 | 22 | 1.8 | 1 | 1 | 1 | 7 | 12 | 1.7 | 2 | 2 | 1 | 10 | 64 | 6.4 |
| M11 | 8 | 16 | 2.0 | 16 | 24 | 1.5 | 0 | 0 |  | 7 | 15 | 2.1 | 5 | 5 | 1 | 8 | 71 | 8.9 |
| M12 | 8 | 16 | 2.0 | 11 | 16 | 1.5 | 0 | 0 |  | 9 | 16 | 1.8 | 1 | 1 | 1 | 12 | 70 | 5.8 |
| M13 | 9 | 19 | 2.1 | 16 | 28 | 1.8 | 0 | 0 |  | 7 | 17 | 2.4 | 1 | 1 | 1 | 11 | 74 | 6.7 |
| M14 | 7 | 15 | 2.1 | 19 | 26 | 1.4 | 0 | 0 |  | 7 | 12 | 1.7 | 4 | 4 | 1 | 12 | 59 | 4.9 |
| M15 | 7 | 16 | 2.3 | 22 | 34 | 1.5 | 1 | 1 | 1 | 8 | 18 | 2.3 | 3 | 3 | 1 | 11 | 64 | 5.8 |
| M16 | 10 | 18 | 1.8 | 16 | 22 | 1.4 | 0 | 0 |  | 7 | 13 | 1.9 | 1 | 1 | 1 | 12 | 64 | 5.3 |
| M17 | 8 | 18 | 2.3 | 11 | 17 | 1.5 | 1 | 1 | 1 | 6 | 12 | 2.0 | 2 | 2 | 1 | 11 | 65 | 5.9 |
| M18 | 8 | 16 | 2.0 | 11 | 18 | 1.6 | 1 | 1 | 1 | 5 | 9 | 1.8 | 5 | 5 | 1 | 15 | 67 | 4.5 |
| M19 | 8 | 16 | 2.0 | 18 | 32 | 1.8 | 0 | 0 |  | 6 | 11 | 1.8 | 1 | 1 | 1 | 12 | 66 | 5.5 |
| M20 | 6 | 14 | 2.3 | 13 | 19 | 1.5 | 0 | 0 |  | 7 | 13 | 1.9 | 2 | 2 | 1 | 10 | 67 | 6.7 |

| PID -> | U5678295899 | | | U8459342968 | | | U7830411862 | | | U6787331075 | | | U4495597388 | | | U2428440101 | | |
| --- | --- | --- | --- | --- | --- | --- | --- | --- | --- | --- | --- | --- | --- | --- | --- | --- | --- | --- |
|  |  | | |  | | |  | | |  | | |  | | |  | | |
| Model # | Frequency of episodes | DAYS | average duration of episode in days | Frequency of episodes | DAYS | average duration of episode in days | Frequency of episodes | DAYS | average duration of episode in days | Frequency of episodes | DAYS | average duration of episode in days | Frequency of episodes | DAYS | average duration of episode in days | Frequency of episodes | DAYS | average duration of episode in days |
| M0 | 1 | 1 | 1 | 1 | 1 | 1.0 | 0 | 0 |  | 4 | 20 | 5.0 | 2 | 2 | 1 | 10 | 20 | 2.0 |
| M1 | 4 | 4 | 1 | 5 | 6 | 1.2 | 1 | 1 | 1 | 37 | 166 | 4.5 | 4 | 4 | 1 | 30 | 54 | 1.8 |
| M2 | 2 | 2 | 1 | 2 | 2 | 1.0 | 0 | 0 |  | 27 | 202 | 7.5 | 3 | 3 | 1 | 25 | 43 | 1.7 |
| M3 | 1 | 1 | 1 | 1 | 1 | 1.0 | 0 | 0 |  | 30 | 175 | 5.8 | 3 | 3 | 1 | 38 | 60 | 1.6 |
| M4 | 1 | 1 | 1 | 2 | 3 | 1.5 | 2 | 2 | 1 | 33 | 188 | 5.7 | 3 | 3 | 1 | 21 | 37 | 1.8 |
| M5 | 1 | 1 | 1 | 3 | 4 | 1.3 | 0 | 0 |  | 34 | 173 | 5.1 | 4 | 4 | 1 | 32 | 51 | 1.6 |
| M6 | 1 | 1 | 1 | 3 | 3 | 1.0 | 0 | 0 |  | 32 | 203 | 6.3 | 6 | 6 | 1 | 22 | 37 | 1.7 |
| M7 | 3 | 3 | 1 | 3 | 3 | 1.0 | 1 | 1 | 1 | 37 | 144 | 3.9 | 3 | 3 | 1 | 27 | 49 | 1.8 |
| M8 | 2 | 2 | 1 | 1 | 1 | 1.0 | 0 | 0 |  | 33 | 176 | 5.3 | 3 | 3 | 1 | 28 | 49 | 1.8 |
| M9 | 1 | 1 | 1 | 3 | 3 | 1.0 | 2 | 2 | 1 | 26 | 203 | 7.8 | 4 | 4 | 1 | 22 | 37 | 1.7 |
| M10 | 3 | 3 | 1 | 5 | 5 | 1.0 | 0 | 0 |  | 28 | 199 | 7.1 | 2 | 2 | 1 | 24 | 46 | 1.9 |
| M11 | 2 | 2 | 1 | 10 | 13 | 1.3 | 0 | 0 |  | 37 | 160 | 4.3 | 4 | 4 | 1 | 32 | 52 | 1.6 |
| M12 | 1 | 1 | 1 | 5 | 5 | 1.0 | 1 | 1 | 1 | 25 | 209 | 8.4 | 5 | 5 | 1 | 26 | 42 | 1.6 |
| M13 | 2 | 2 | 1 | 1 | 1 | 1.0 | 0 | 0 |  | 31 | 197 | 6.4 | 2 | 2 | 1 | 23 | 38 | 1.7 |
| M14 | 2 | 2 | 1 | 6 | 6 | 1.0 | 0 | 0 |  | 29 | 191 | 6.6 | 2 | 2 | 1 | 35 | 58 | 1.7 |
| M15 | 1 | 1 | 1 | 2 | 2 | 1.0 | 0 | 0 |  | 21 | 216 | 10.3 | 2 | 2 | 1 | 26 | 38 | 1.5 |
| M16 | 1 | 1 | 1 | 3 | 3 | 1.0 | 2 | 2 | 1 | 35 | 189 | 5.4 | 3 | 3 | 1 | 29 | 49 | 1.7 |
| M17 | 1 | 1 | 1 | 9 | 14 | 1.6 | 0 | 0 |  | 36 | 184 | 5.1 | 3 | 3 | 1 | 28 | 48 | 1.7 |
| M18 | 1 | 1 | 1 | 3 | 4 | 1.3 | 0 | 0 |  | 34 | 186 | 5.5 | 4 | 4 | 1 | 35 | 61 | 1.7 |
| M19 | 1 | 1 | 1 | 1 | 1 | 1.0 | 0 | 0 |  | 29 | 196 | 6.8 | 3 | 3 | 1 | 20 | 33 | 1.7 |
| M20 | 2 | 2 | 1 | 3 | 3 | 1.0 | 3 | 3 | 1 | 24 | 199 | 8.3 | 2 | 2 | 1 | 32 | 51 | 1.6 |

| PID -> | U0366211537 | | | |
| --- | --- | --- | --- | --- |
|  |  | | | |
| Model # | Frequency of episodes | DAYS | average duration of episode in days | |
| M0 | 10 | 18 | 1.8 |  |
| M1 | 48 | 120 | 2.5 |  |
| M2 | 37 | 82 | 2.2 |  |
| M3 | 45 | 141 | 3.1 |  |
| M4 | 44 | 98 | 2.2 |  |
| M5 | 36 | 85 | 2.4 |  |
| M6 | 38 | 87 | 2.3 |  |
| M7 | 31 | 65 | 2.1 |  |
| M8 | 30 | 74 | 2.5 |  |
| M9 | 42 | 78 | 1.9 |  |
| M10 | 44 | 111 | 2.5 |  |
| M11 | 53 | 123 | 2.3 |  |
| M12 | 36 | 85 | 2.4 |  |
| M13 | 42 | 87 | 2.1 |  |
| M14 | 40 | 85 | 2.1 |  |
| M15 | 40 | 79 | 2.0 |  |
| M16 | 36 | 73 | 2.0 |  |
| M17 | 47 | 95 | 2.0 |  |
| M18 | 43 | 98 | 2.3 |  |
| M19 | 51 | 131 | 2.6 |  |
| M20 | 47 | 121 | 2.6 |  |

Table S2 – Bhopal site – comparison of demographic characteristics of the full (n=22) and analytic sample (n=14)

Note: Out of the full sample, based on the presence of one or more instances of SI and the daily survey usage rate cut-off for the site, the 14 participants were selected for analysis.

| Demographic characteristic | Full sample (n=22) | Analytic sample (n=14) |
| --- | --- | --- |
|  |  |  |
| Average Age | 29 years | 30.8 years |
| Age range (years) | 21-42 | 23-42 |
| Gender Distribution | Male: 60%, Female:40% | Male: 50%, Female:50% |
| Average years of education | 12.8 | 11.8 |
| Percentage married | 10% | 16.7% |
| Percentage single | 90% | 83.3% |

Table S3 – Bengaluru site – comparison of demographic characteristics of the full (n=22) and analytic sample (n=14)

| Demographic characteristic | Full sample (n=22) | Analytic sample (n=14) |
| --- | --- | --- |
|  |  |  |
| Average Age | 31.6 years | 33.9 years |
| Age range (years) | 18-42 | 31-42 |
| Gender Distribution | Male: 66.7%, Female: 33.3% | Male: 62.5%, Female: 37.5% |
| Average years of education | 13.8 | 13.6 |
| Percentage married | 8.33% | 12.5% |
| Percentage single | 91.67% | 87.5% |
